# Service Users' Perspectives on Communicating Compassion in Mental Health Practice

**DOI:** 10.1002/nop2.70081

**Published:** 2024-11-05

**Authors:** Ellie Wildbore, Carmel Bond, Stephen Timmons, Ada Hui, Shane Sinclair

**Affiliations:** ^1^ Sheffield Health and Social Care NHS Foundation Trust Sheffield UK; ^2^ School of Nursing and Midwifery Sheffield Hallam University Sheffield UK; ^3^ Centre for Health Innovation, Leadership and Learning, Nottingham University Business School University of Nottingham Nottingham UK; ^4^ Royal College of Nursing London UK; ^5^ Compassion Research Lab, Faculty of Nursing University of Calgary Calgary Alberta Canada

**Keywords:** clinical skills, communication, compassion, engagement, nursing practice, service users

## Abstract

When people talk about their healthcare experience, compassion is often a common ingredient in the stories they share. After a decade of healthcare reforms and research on compassion, the experience of receiving compassionate care has been shown to be important to patients and their families. Yet, there is little guidance to inform compassionate practice in the context of providing mental health care. In this article, the authors suggest three things that mental health nurses can use in their practice to demonstrate compassion.

## 1 Evidence

That's what everything comes down to…if someone is there and they are compassionate that can be just so healing

The above quote is from a mental health service user who took part in our recent study (Bond, Hui, Timmons, and Wildbore, et al. 2022) where we asked about compassion in the care they had received. Compassion is a word that is often dismissed, yet when people talk about their experiences of care, it is compassion (or a lack of compassion) that is a central narrative in the stories people share. However, it is no longer these simple anecdotal accounts that demonstrate that compassion is what service users *really* want. There is now a convincing amount of research evidence confirming the importance of compassion in health care.

Compassion in health care has been studied for over a decade and has been found to be one of the most important aspects of quality care provision. Compassion has been shown to significantly improve service users' satisfaction with their experiences of care and is associated with improved perceptions of quality care by the people who receive it (Sinclair et al. 2017; Taylor, LeBlanc, and Nosik 2019). It is at the heart of the professional guidelines for nurses and is thought to be essential for the delivery of safe, effective nursing practice (Nursing and Midwifery Council 2018).

If you look around the NHS, compassion seems to be everywhere. It is recognised as a fundamental nursing value, central to the NHS Constitution (England 2015), and is frequently cited in organisational vision statements. But what is compassion *really*? How do you know when you deliver compassion in your professional practice? When you think about the word, ‘compassion’, what images does it bring to mind? What does it look like? What does it feel like when you receive compassion? Perhaps in thinking about this, you thought about a time when you received health care. Perhaps you have been the one using a service, or you took a family member to an appointment. What was it about that particular experience that left a lasting impression?

In the context of receiving mental health care, we suggest that compassion is of particular significance. We have spoken to mental health nurses, who told us that compassion is a part of their professional identity (Bond, Hui, Timmons, and Charles, et al. 2022). This is key because much of what mental health nurses do is expressed through therapeutic relationships, or through ordinary everyday interactions, where individual nursing values can be displayed. We believe that this is where compassion has the opportunity to flow.

Think about this question for a moment. What do mental health nurses have in their metaphorical toolbox to support the recovery process? There are medications, psychological therapies, the therapeutic relationship, and day‐to‐day interactions. We do not deny the potential beneficial effects of medications and/or psychological therapies as these things can be compassionate in of themselves (Nursing and Midwifery Council 2018), however, service users told us they also need to be delivered with compassion. The idea that there are other forms of healing, beyond strictly a medicalised approach that can improve health outcomes, is gaining popularity among academics who are interested in the study of mental health recovery (Hammarström et al. 2020; Von Peter et al. 2019; Spandler and Poursanidou 2019; Gilbert 2010). What we are suggesting here is not a new idea either. Fogarty et al. (1999), showed that just 40 seconds of compassion has the power to reduce anxiety, which is important across all healthcare contexts (Fogarty et al. 1999). There are, however, many perspectives out there regarding what compassion is and how compassion can be communicated. Here, we refer specifically to how compassion has been identified by people who access (or attempt to access) mental health services.

**FIGURE 1:**
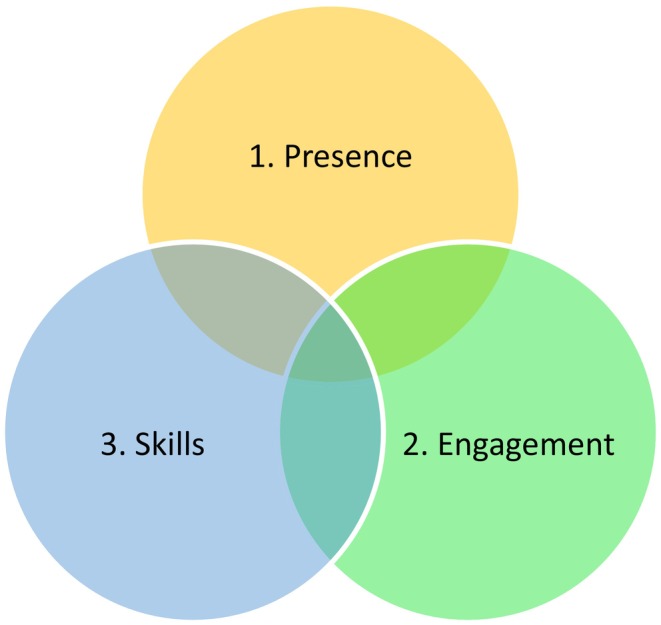
The core, interconnected, components of communicating compassion in mental health practice.

In our recent study (Bond, Hui, Timmons, and Wildbore, et al. 2022), the services users we spoke to outlined three core components to the way mental health nurses communicate compassion in their practice (Figure 1).

1. Presence: This component of communicating compassion is linked to the nurse's presence and the way in which they enter the interaction with the service user.

Service users described nurses as ‘compassionate’ when they displayed a warm, calm, presence. As we previously mentioned, our research participants confirmed that they felt they could feel compassion in their nurse's presences or demeanour within the first few seconds of meeting them (Fogarty et al. 1999).

According to our research participants, the most important thing, in terms conveying a compassionate presence, is to let the service user know that they can trust you. When someone is unwell, entering the interaction in a calm manner provides the service user with the feeling that you are there for them—no matter how the service user is presenting to you. In the context of developing a therapeutic relationship between nurse and service users, this early display of warmth and calmness will enable you to build trust right from the start, which is important for building a therapeutic relationship.

2. Engagement: This component of communicating compassion is linked to the humanistic skills that are fundamental to mental health nursing care, such as working in a non‐judgemental way, and seeking to understand the person hollistically.

There was a very clear message from the participants we spoke to that using a strengths‐based approach was viewed as communicating compassion when engaging with service users. This means, rather than focusing on any deficits (e.g., emphasis on symptoms or psychiatric diagnosis), the focus should be on helping service users to realise their own personal strengths. This could include reminding someone that they are more than their illness or mental health problem, which can help someone to see beyond their diagnosis.

Our participants also told us about feeling vulnerable to ‘put themselves out there’. They referred to experiences where they had relayed their story, in what they felt was a therapeutic space, and were sometimes met with judgemental responses. This led them to disengage from the service.

Compassion was, however, about asking questions to gain clarity, and for the nurse to develop a greater understanding of the person's experiences in the context of their life. Critically, in order to display compassion, service users told us that the nurse must at least attempt to put their own perspective to the side and attempt to see beyond the person's diagnosis. If after the nurse seeks to understand by looking beyond the diagnosis, but then uses this information to make negative assumptions, this is not compassionate. When the nurse was perceived to have used the information to judge the patient, the service user's mental health was adversely affected. For example, when a service user shares their story or feelings, but the nurse judges them based on physical appearance and decides they don't need care. This assessment process, with a focus on ‘objectively’ observed measurements, such as physical appearance, rejects lived experiences.

In contrast, engagement with a person on a human‐to‐human level was felt to embody a compassionate approach. For example, our participants spoke about compassion being related to ‘how people make you feel’ and being treated as a ‘real’ person. This involved the nurse taking time to try, as much as possible, to think about what might be right for this person, and was tied closely to working with someone in a strength‐based way.

While our participants gave examples of what compassionate care meant to them, they also provided multiple examples of having engagements that were not compassionate. Service users (and research evidence) (Bond, Hui, Timmons, and Wildbore, et al. 2022) were clear that a lack of compassion had a serious, negative impact on their mental health and well‐being. This reminds us as nurses and/or healthcare professionals that we need to exercise caution and lead with the desire to understand the person when engaging with our service users, less we enact the opposite of the care ethic of ‘do no harm’ (Nursing and Midwifery Council 2018) and exacerbate their suffering.

3. Skills: This core component of communicating compassion is linked to the nurse or healthcare professionals' clinical skills, such as active listening and validation, which can provide a sense of genuine care.

From the perspective of our participants, mental health nurses need to possess active listening skills and know how to effectively validate a person's feelings and emotions. These skills help the nurse to understand the person's distress and how that might be affecting them personally in the context of their life. Service users told us that this can be as simple as listening to someone's story even if you don't fully understand how they might feel.

Validation and active listening are specific clinical skills that need to be practiced and developed over time. However, changes to the standards of proficiency for nurses (Taylor, LeBlanc, and Nosik 2019) have been criticised for diluting these skills that are believed to be vital to the delivery of mental health care (Haslam 2023). Our participants were clear; actively listening and validation of the person's lived experiences were central to the delivery of compassionate care.

We would go so far as to contend that compassion is a ‘safety critical’ element of mental health nursing because the therapeutic relationship is at the heart of what mental health nurses do. It is what makes mental health nursing unique and offers a distinct compassionate professional identity (Bond, Hui, Timmons, and Charles, et al. 2022).

Compassion is at the core of mental health nursing practice, and it goes beyond trying to work out what it might be like to be in someone else's shoes (Sinclair et al. 2017, 2018). Compassion is about going the extra mile to *really* understand what a person might need and how those needs might fluctuate as a person's recovery journey or personal trajectory changes. Our research on compassion in mental health, from the perspective of individuals who access services, resonates with a much wider body of work on compassion (Malenfant et al. 2022). Collectively, these findings highlight how important it is that nurses (and aspiring nurses) develop the skills that are linked to compassion in mental health nursing. Arguably, these interpersonal skills, linked to compassion, are necessary for all fields of nursing. It is crucial to be mindful that, while thinking about what compassion looks like and feels like from your personal perspective, many of the people mental health nurses encounter have experienced trauma. So, the ways in which you might imagine compassion to be expressed (e.g., through physical touch) might not be the same for someone else, and could, in fact, cause more harm than good. The most important aspect of expressing compassion in mental health nursing, therefore, is to seek to understand the person, who ‘they’ are, and what has happened to them, and what they actually need (Sweeney et al. 2018).

## 2 Closing Remarks

People who access mental health services often need the help of nurses and healthcare professionals to develop coping skills and begin to process what has happened in their lives. It is on this basis we encourage you to consider how you might be that person who makes a lasting impression by offering everyone you come into contact with the compassion they expect and may desperately need.

## Conflicts of Interest

The authors declare no conflicts of interest.

## Data Availability

The authors have nothing to report.
